# Indian Clinicians’ Perspectives on Utilizing Dapagliflozin-Based Combination Therapy for the Management of Obesity in Patients With Type 2 Diabetes Mellitus

**DOI:** 10.7759/cureus.92594

**Published:** 2025-09-17

**Authors:** Mayur Agrawal, Abhay Ahluwalia, Raman Boddula, Arjun Baidya, Hiteswar Saikia, Anu Mathew, Prem Naryanan, Arjun R, Modhugu N Reddy, Rohan N Kesarkar, Abhijit Pednekar

**Affiliations:** 1 Endocrinology and Diabetes, Hormone India Diabetes and Endocrine Center, Bhopal, IND; 2 Endocrinology, Dr. Abhay Ahluwalia Diabetes and Endocrine Clinic, Gurugram, IND; 3 Endocrinology, Diabetes and Metabolism, Yashoda Hospitals, Secunderabad, IND; 4 Endocrinology, Nil Ratan Sarkar Medical College and Hospital, Kolkata, IND; 5 Endocrinology, Jorhat Medical College and Hospital, Jorhat, IND; 6 Diabetes and Endocrinology, Fortis Hospital, Gurugram, IND; 7 Endocrinology, Ahalia Diabetes Hospital, Palakkad, IND; 8 Endocrinology and Diabetes, Aster Malabar Institute of Medical Sciences (MIMS) Hospital, Kannur, IND; 9 Endocrinology and Diabetes, Asian Institute of Gastroenterology (AIG) Hospitals, Hyderabad, IND; 10 Diabetes and Endocrinology, Scientific Services, USV Pvt Ltd., Mumbai, IND; 11 Scientific Services, USV Pvt Ltd., Mumbai, IND

**Keywords:** diabetes mellitus type 2, heathcare surveys, metformin, obesity, sodium-glucose cotransporter-2 inhibitors

## Abstract

Background: In India, abdominal obesity is highly prevalent among both men and women and is associated with an increased risk of developing type 2 diabetes mellitus (T2DM). This presents a dual challenge for Indian clinicians in simultaneously managing body weight and glycemic levels. This article aimed to understand Indian clinicians' perspectives regarding obesity and T2DM association and utilization of dapagliflozin+metformin combination therapy for obesity management in patients with T2DM.

Methods: A cross-sectional online survey was conducted with 914 Indian clinicians, including endocrinologists, diabetologists, consultants, and general physicians. A structured questionnaire was used to evaluate clinicians' knowledge of the role of obesity in T2DM, their attitudes toward weight management, and current practices in addressing obesity in patients with T2DM. The survey also assessed clinicians' utilization of pharmacological and nonpharmacological interventions, and their perceptions of barriers to effective obesity management. Data from the questionnaires were analyzed using Statistical Package for the Social Sciences version 29.0 (IBM Corp., Armonk, NY). Descriptive and inferential statistics were also calculated. Statistical significance was set at p< 0.05.

Results: Of the 914 clinicians, 910 (99.50%) agreed that weight management and glycemic control in T2DM patients are the primary treatment goals. Cardiovascular disease and hypertension were identified as the most recognized complications of obesity in T2DM patients by 742 (81.80%) and 682 (74.61%) clinicians, respectively. Dapagliflozin+metformin fixed-dose combinations (FDCs) were the preferred initial glucose-lowering combination for overweight/obese T2DM patients by 624 (68.28%) clinicians, followed by dapagliflozin+sitagliptin FDCs by 390 (42.68%) clinicians. Glycemic control, weight loss, and cardiovascular and renal benefits are critical considerations for clinicians. A majority of 670 (73.30%) clinicians reported noncompliance, followed by 570 (62.36%) who reported pill burden as a significant barrier to managing obesity in patients with T2DM.

Conclusion: Most Indian clinicians recognize the importance of obesity management in patients with T2DM. However, patient noncompliance and pill burden were identified as significant barriers to managing obesity in these patients. There is a preference for dapagliflozin+metformin FDCs in the management of overweight T2DM patients. Thus, prescribing FDCs in routine clinical practice and ensuring patient access could improve the outcomes in this high-risk group.

## Introduction

Obesity is a significant global public health concern, particularly in patients with type 2 diabetes mellitus (T2DM) [1]. Obesity significantly influences the development, progression, and associated complications of T2DM [1]. The World Health Organization (WHO) states that in 2022, 2.5 billion adults aged 18 years and older were overweight, including over 890 million with obesity. This corresponds to 43% of adults (43% of men and 44% of women) being overweight, up from 25% in 1990. Being overweight in childhood and adolescence affects immediate health and is linked to a higher risk and earlier onset of noncommunicable diseases (NCDs) such as T2DM and cardiovascular diseases (CVDs) [2]. A report by the American Diabetes Association (ADA) confirms earlier predictions regarding the epidemic nature of T2DM globally during the first quarter of the 21st century. The global prevalence of T2DM among adults is projected to rise to 300 million by the year 2025, with a higher incidence among middle-aged adults than among elderly adults. These data suggest that age alone does not completely account for this burden. Instead, variations in critical lifestyle-related risk factors, such as unhealthy dietary habits and physical inactivity, may contribute to the development of central obesity, which is a significant determinant of T2DM [3]. The term "diabesity” indicates that most T2DM patients are overweight or obese [4]. Data from a nationwide cluster survey (Niyantrita Madhumeha Bharata 2017) of 100,531 adults showed a 40.3% prevalence of obesity in India. The south showed the highest (46.51%) and the east the lowest (32.96%). Obesity was higher in women (41.88%), urban areas (44.17%), and those over 40 years of age (45.81%) [5]. The 2019-21 National Health Survey in India showed an abdominal obesity prevalence of 51.77% and 57.91% among men and women, respectively. In both genders, abdominal obesity was associated with T2DM, with 27% and 5% higher odds among those with abdominal obesity, respectively. A significant interaction exists between abdominal obesity and high body mass index (BMI) regarding T2DM odds in the Indian population [6]. With this epidemiological disease burden, the economic burden of obesity and T2DM in India is significant, resulting in an annual expenditure of $23.2 billion, which constitutes 1.7% of the nation's gross domestic product. Projections suggest that this could culminate in a loss of $440 billion by 2060 [7]. Thus, addressing the obesity epidemic in India is imperative for the prevention of T2DM.

Obesity and ectopic fat accumulation are vital pathogenic factors that cause insulin resistance (IR) in patients with T2DM [8]. Free fatty acids mediate IR and glucose tolerance and are linked to central obesity. Alpha-ketoglutarate-dependent dioxygenase (FTO) is associated with an increased risk of obesity and T2DM in Indians. The adenosine triphosphate-binding cassette subfamily C member 8 gene poses a risk to European and Indian populations [9]. Thus, achieving both weight loss and glycemic targets is crucial for T2DM treatment. As a primary treatment for T2DM, lifestyle-induced weight loss significantly improves glycemic control and the associated risk factors. T2DM patients who lose weight are more likely to achieve target glycated hemoglobin (HbA1c) levels than those who maintain or gain weight [10]. A meta-analysis of obesity T2DM patients revealed that losing more than 5% of body weight substantially reduces fasting blood glucose, lipid levels, and blood pressure (BP) [11]. A ≥3% weight loss improved BP, fasting blood glucose, and cholesterol levels. The microvascular complications of T2DM, such as retinopathy, nephropathy, and peripheral and autonomic neuropathy, are influenced by the duration of diabetes and obesity [10,11].

The consensus statement by the American Association of Clinical Endocrinologists, the American College of Endocrinology on Type 2 Diabetes Management Algorithm, and the American Diabetes Association Standards of Medical Care recommends weight reduction through lifestyle and pharmacological interventions for managing obesity and T2DM. However, many glucose-lowering therapies, such as insulin, sulfonylureas, glinides, and thiazolidinediones, cause weight gain. Recently introduced glucagon-like peptide-1 receptor agonists (GLP-1 RAs) and sodium-glucose cotransporter-2 inhibitors (SGLT2i), which promote weight loss, are now preferred for managing T2DM patients with overweight/obesity [12,13]. A recent meta-analysis indicated that dapagliflozin at 5 mg/day (a commonly used SGLT2i) exhibited a more significant weight loss effect [14] and, when combined with conventional antidiabetic drugs (metformin and metformin/sitagliptin), improved glycemic control and reduced weight gain in patients with T2DM [15]. A multicenter prospective noninterventional observational real-world evidence (FOREFRONT) study in India observed a significant reduction in HbA1c levels and body weight in T2DM patients on dapagliflozin. The mean (SD) of HbA1c levels demonstrated a significant reduction from the baseline measurement of 9.11% (1.44) to 8.11% (1.22) at three months and further decreased to 7.62% (1.04) at six months, with p < 0.001 [16]. Another electronic medical record-based, retrospective, multicenter Indian study found that dapagliflozin significantly improved glycemic parameters and BMI when added to metformin in a real-world scenario [17]. The ADA, Research Society for Study of Diabetes in India, Endocrine Society of India, and Asian Perspective and Expert Recommendations suggest SGLT2i over other oral hypoglycemic agents (OHAs) for managing T2DM patients with CVDs, chronic kidney disease (CKD), and obesity [18-20].

The approach to managing obesity in patients with T2DM has evolved, and SGLT2i, including dapagliflozin and its combination therapies, have emerged as a viable treatment option. However, addressing obesity and T2DM requires a comprehensive and integrated approach involving various healthcare professionals (HCPs). Knowledge, attitudes, and practices (KAPs) of HCPs regarding obesity management in T2DM patients are vital, as they are the primary source of health information [21,22]. A nationwide study in India by Bafna et al. [23] examined clinicians' views on the association between heart failure (HF) and T2DM in Indian patients and their clinical practices concerning SGLT2i usage, such as dapagliflozin, for cardiorenal protection in T2DM patients. Limited studies exist on Indian clinicians' perceptions regarding the challenges of obesity management in T2DM patients and their views on prescribing dapagliflozin-based fixed-dose combinations (FDCs). This study aimed to evaluate clinicians' perceptions of obesity and T2DM management and assess dapagliflozin-based FDCs' prescribing patterns in this high-risk patient group.

## Materials and methods

An anonymous, cross-sectional, noninterventional PAN India survey was conducted between January 15, 2024, and March 15, 2024. Clinicians from various regions of India with expertise in T2DM management were included. Convenience sampling was used to recruit 920 clinicians, including diabetologists, cardiologists, endocrinologists, consultant physicians, general practitioners, and other medical practitioners. No restrictions were placed on participation based on years of professional experience. The initial page of the web-based questionnaire explained the survey's objectives and emphasized its anonymous nature. As the survey involved voluntary, anonymous participation of HCPs and did not involve patient data or interventions, ethical approval was not mandatory under the Indian guidelines (Indian Council of Medical Research, 2017) [24]. The study adhered to the principles of the Declaration of Helsinki, including revisions and regulations for data management and confidentiality. A survey response from clinicians was considered consent to participate.

Survey instrument

A structured questionnaire was developed to collect data on clinicians’ management practices for obesity in T2DM patients. It was developed using a rigorous process that involves several steps. A thorough review of existing literature on T2DM management, particularly in obesity patients, was conducted to identify questions relevant to the survey. Input from experts in obesity management in T2DM patients was considered to ensure the validity and relevance of the questionnaire.

Definition and classification of obesity

Obesity

The Lancet Commission defines obesity as a condition characterized by excess adiposity, with or without abnormal distribution or function of adipose tissue, and with multifactorial causes that are still incompletely understood [25].

Clinical Obesity

Clinical obesity is defined as a chronic, systemic illness characterized by alterations in the function of tissues, organs, the entire individual, or a combination thereof, due to excess adiposity. Clinical obesity can lead to severe end-organ damage, causing life-altering and potentially life-threatening complications (e.g., heart attack, stroke, and renal failure) [25].

Preclinical Obesity

Preclinical obesity is defined as a state of excess adiposity with preserved function of other tissues and organs and a varying but generally increased risk of developing clinical obesity and several other NCDs (e.g., type 2 diabetes, CVD, certain types of cancer, and mental disorders) [25].

Abdominal/Visceral Obesity

Abdominal/visceral obesity, also known as central obesity, is considered a more serious form of fat distribution as it predisposes individuals to various metabolic disorders and diseases [26].

Abdominal Obesity

Abdominal obesity is defined as a waist circumference >80 cm in women and >94 cm in men [26].

The BMI is the most commonly used metric for evaluating body weight. It is calculated by dividing the patient's weight by the square of their height (kg/m²). The BMI stratifies patients into the following categories: 1) underweight: less than 18.5 kg/m²; 2) healthy weight: 18.5-24.9 kg/m² (18.5-22.9 kg/m² in Asians); 3) overweight: 25-29.9 kg/m² (23-27.4 kg/m² in Asians); 4) obesity, class 1: 30-34.9 kg/m² (27.5-32.4 kg/m² in Asians); 5) obesity, class 2: 35-39.9 kg/m² (32.5-37.4 kg/m² in Asians); 6) obesity, class 3: >40 kg/m² (greater than 37.5 kg/m² in Asians); 7) the upper value in each category is lower for Asians due to higher body fat and increased T2DM risk at a lower BMI. Many international societies have adopted these lower ranges for obesity in Asians [27-29]; and 8) WHO recommended BMI cutoff for categorization: underweight (<18.5 kg/m²), normal BMI (18.5 to <25 kg/m²), overweight (25 kg/m² to <30 kg/m²), and obesity (≥30 kg/m²) [27-29].

Questionnaire content

The questionnaire focused on the knowledge, attitudes, and practices of Indian clinicians regarding weight management in managing obesity in T2DM patients, adherence to clinical practice guidelines related to obesity and T2DM, barriers to providing optimal care, and clinical scenarios involving overweight T2DM patients (Appendix 1). These scenarios evaluated how clinicians would approach obesity management using the preferred OHAs in patients with T2DM. Questions 1-4 evaluated clinicians’ knowledge regarding obesity and poor glycemic control, adherence to obesity and T2DM treatment guidelines, and comorbidities associated with obesity in T2DM patients. The subsequent three questions (Questions 5-7) examined clinicians' attitudes toward weight management for obesity T2DM patients, including the prescription of dapagliflozin, metformin FDC, and lifestyle modifications. The final section (Questions 8-13) assessed clinicians' practical approaches, including daily patient load, challenges in managing obesity in T2DM patients, factors guiding their medication choices regarding various glucose-lowering agents, and specific clinical scenarios in which they would or would not prescribe specific pharmacological therapies (such as dapagliflozin+metformin, dapagliflozin+sitagliptin, or dapagliflozin+metformin+sitagliptin FDCs).

Survey dissemination

A pilot study was conducted among 10 clinicians to assess the clarity, relevance, and comprehensiveness of the questionnaire items and to provide comments to determine the survey's content and face validity. The final questionnaire comprised 13 open- and closed-ended questions. The final survey questionnaire was disseminated via an online link to the clinicians' registered email addresses. The study included a URL that directed recipients to an online version of the questionnaire created and hosted using Google Forms (Google LLC, Mountain View, CA). The participants either completed and returned the survey via email or completed the digital version online.

Survey assessment

A Likert scale assessed clinicians’ KAP regarding obesity management in T2DM patients. The Likert scale consisted of a five-point categorical response scale: “Strongly Agree,” “Agree,” “Undecided,” “Disagree,” and “Strongly Disagree.” This response format allowed clinicians to indicate their agreement or disagreement with statements/questions regarding obesity management in T2DM patients. The Likert scale items were carefully crafted to cover multiple topics, including perceptions of current management strategies, barriers to optimal care delivery, and attitudes toward managing obesity in T2DM patients. Each item presented a statement or assertion relevant to the study objectives, and the participants were required to select the response option that best reflected their agreement or disagreement with the question. The Likert-scale responses were then coded numerically for quantitative analysis, with higher scores indicating a more significant agreement or alignment.

Statistical analysis

Data were extracted from the questionnaires, transferred to a Microsoft Excel spreadsheet (Microsoft Corporation, Redmond, WA), and analyzed using Statistical Package for the Social Sciences (SPSS) version 29.0 (IBM Inc., Chicago, IL). Descriptive and inferential statistics were also calculated. Categorical variables are presented as proportions and frequencies, whereas continuous variables are presented as means and SDs. The Likert scale responses were reported as percentages and frequencies. Statistical significance was set at p < 0.05.

## Results

In total, 914 clinicians who completed the questionnaires were included in the final analysis.

Clinical characteristics of survey participants

Of the 914 Indian clinicians surveyed, 277 (30.31%) were from the southern zone of India, 184 (20.13%) from the western zone, 161 (17.61%) from the northern zone, 160 (17.51%) from the eastern zone, and 132 (14.44%) from the central zone. The central zone included Chhattisgarh, Uttar Pradesh, and Madhya Pradesh. The east zone included Odisha, West Bengal, Bihar, Manipur, and Jharkhand. The north zone included Himachal Pradesh, Rajasthan, Delhi, Haryana, Punjab, and Uttarakhand. The south zone included Kerala, Telangana, Tamil Nadu, Karnataka, and Andhra Pradesh. The west zone included Gujarat and Maharashtra. The majority of 505 (55.25%) were consultant physicians, 154 (16.85%) were diabetologists, 142 (15.54%) were cardiologists, 51 (5.58%) were endocrinologists, 55 (6.02%) were general physicians, and seven (0.77%) were clinicians from other specialties (Table 1).

**Table 1:** Distribution of clinicians by zone and speciality (N = 914) n: number of participants; N: total participants ^*^West Bengal: 9; Rajasthan: 7; Delhi: 6; Gujarat: 6; Tamil Nadu: 6; Uttar Pradesh: 4; Andhra Pradesh: 3; Telangana: 3; Odisha: 2; Kerala: 2; Karnataka: 1; Maharashtra: 1; Uttarakhand: 1

Variables	Number of clinicians (n)	Percentage (%)
Zone		
North	161	17.61%
South	277	30.31%
East	160	17.51%
West	184	20.13%
Central region	132	14.44%
Speciality		
Diabetologists	154	16.85%
Cardiologists	142	15.54%
Endocrinologists^*^	51	5.58%
General physicians	55	6.02%
Consultant physicians	505	55.25%
Others	7	0.77%

Knowledge assessment of clinicians on obesity and T2DM

The survey findings demonstrated that 455 (49.78%) clinicians strongly agreed and 426 (46.61%) agreed that overweight T2DM patients had uncontrolled blood glucose levels more often than did normal T2DM patients. For guideline-based clinical practice, 417 (45.62%) clinicians strongly agreed, and 466 (50.98%) agreed that the guidelines recommend that among overweight/obese people living with T2DM, weight management should present a primary goal of treatment along with glycemic management. Of the 914 clinicians surveyed, 859 (93.98%) clinicians either strongly agreed or agreed to recognize the benefits of a 3%-7% reduction in baseline weight for overweight/obesity T2DM patients, demonstrating adherence to clinical practice guidelines (Table 2).

**Table 2:** Clinicians' knowledge assessment on obesity and T2DM (N = 914) T2DM: type 2 diabetes mellitus; n: number of participants; N: total participants; CI: confidence interval; df: degree of freedom, t-test; NR: no response ^*^One-sample t-test

Questions	Strongly agree, n (%)	Agree, n (%)	Undecided, n (%)	Disagree, n (%)	Strongly disagree, n (%)	p value	95% CI	t-statistics^*^	df
Overweight T2DM patients have uncontrolled blood sugar levels more often than normal-weight T2DM patients	455 (49.78%)	426 (46.61%)	18 (1.97%)	15 (1.64%)	NR	<0.001	1.51-1.59	75.723	913
Guidelines recommend that among overweight or obese people living with T2DM, weight management should represent a primary goal of treatment, along with glycemic management	417 (45.62%)	466 (50.98%)	27 (2.95%)	3 (0.33%)	1 (0.11%)	<0.001	1.55-1.62	82.650	913
Guidelines recommend that overweight or obesity people living with T2DM may benefit from a 3%-7% baseline weight loss	341 (37.31%)	518 (56.67%)	44 (4.81%)	11 (1.2%)	NR	<0.001	1.66-1.74	83.413	913

**Figure 1:**
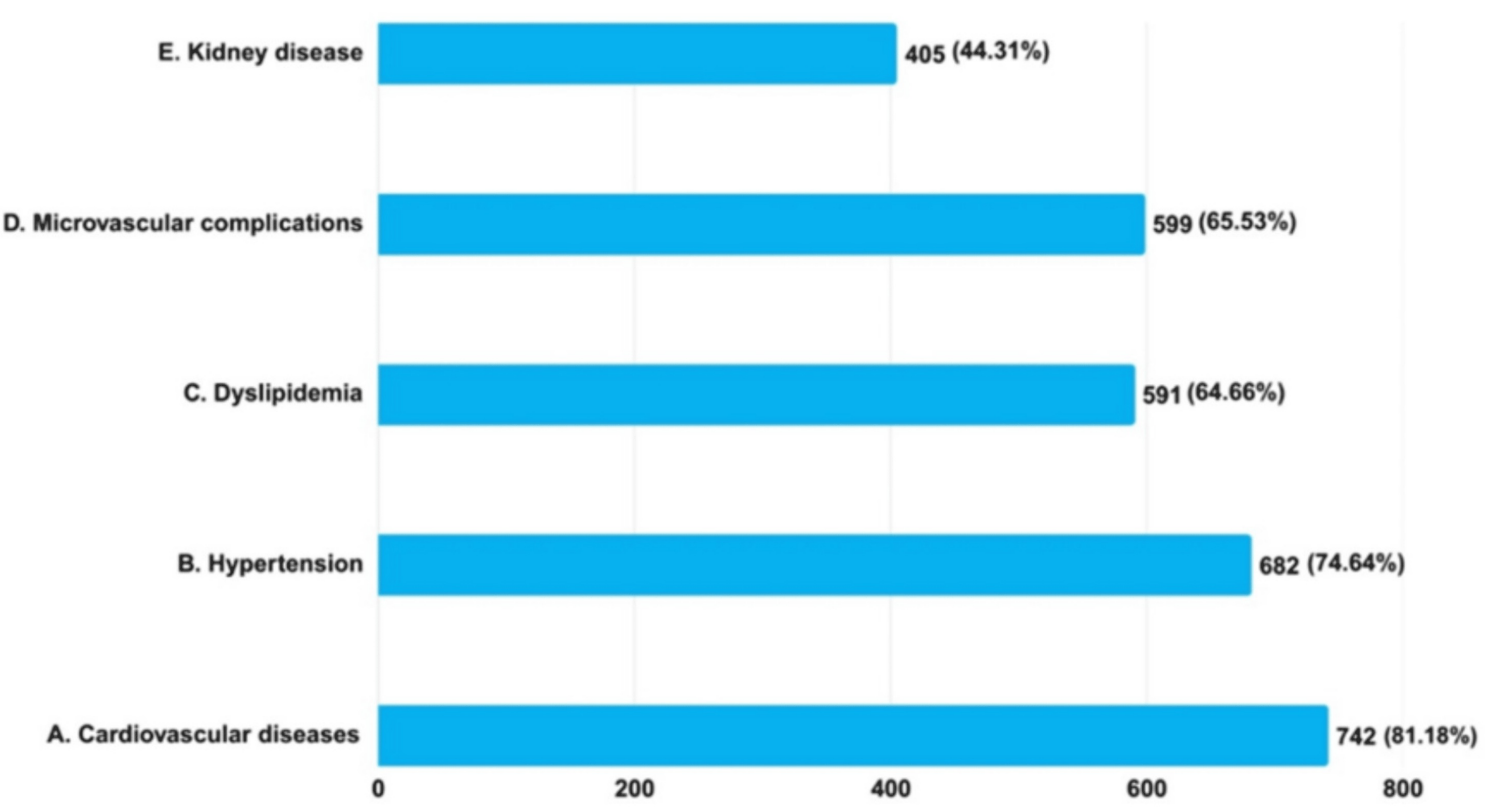
Assessment of clinicians' knowledge regarding major complications associated with obesity in T2DM patients (N = 914) T2DM: type 2 diabetes mellitus

According to the findings, 742 (81.18%) clinicians reported CVDs as a significant complication associated with obesity, 682 (74.61%) reported hypertension, 599 (65.53%) reported microvascular complications, 591 (64.66%) reported dyslipidemia, and 405 (44.31%) reported kidney disease as a significant complication associated with obesity in patients with T2DM (Figure 1).

Attitude assessment of clinicians in managing obesity in T2DM

A substantial majority of 858 (93.87%) clinicians either strongly agreed or agreed that lifestyle interventions effectively manage obesity in T2DM patients. Consequently, 326 (35.67%) strongly agreed, and 541 (59.19%) agreed to prescribe dapagliflozin+metformin FDC as per the most recent Indian real-world evidence (Assessing Dapagliflozin And Metformin As An Initial Regimen In Diabetes Mellitus For Enhanced Glucose Control, ADMIRE, Study), which indicates that dapagliflozin+metformin FDC is mainly preferred for weight loss beyond glucose control (Table 3).

**Table 3:** Clinicians' attitude assessment in managing obesity in T2DM patients (N = 914) CI: confidence interval; FDC: fixed-dose combination; ADMIRE: Assessing Dapagliflozin And Metformin As An Initial Regimen In Diabetes Mellitus For Enhanced Glucose Control; T2DM: type 2 diabetes mellitus; n: number of participants; N: total participants; df: degree of freedom, t-test ^*^One-sample t-test

Questions	Strongly agree, n (%)	Agree, n (%)	Undecided, n (%)	Disagree, n (%)	Strongly disagree, n (%)	p value	95% CI	t-statistics^*^	df
Do you believe that lifestyle interventions are effective in managing obesity in type 2 diabetes patients?	391 (42.78%)	467 (51.09%)	52 (5.69%)	3 (0.33%)	1 (0.11%)	<0.001	1.60-1.68	80.623	913
Most recent Indian real-world evidence (ADMIRE study) indicates that dapagliflozin+metformin FDC is mainly preferred for weight loss beyond glucose control	326 (35.67%)	541 (59.19%)	42 (4.60%)	4 (0.44%)	1 (0.11%)	<0.001	1.66-1.74	88.063	913

**Figure 2:**
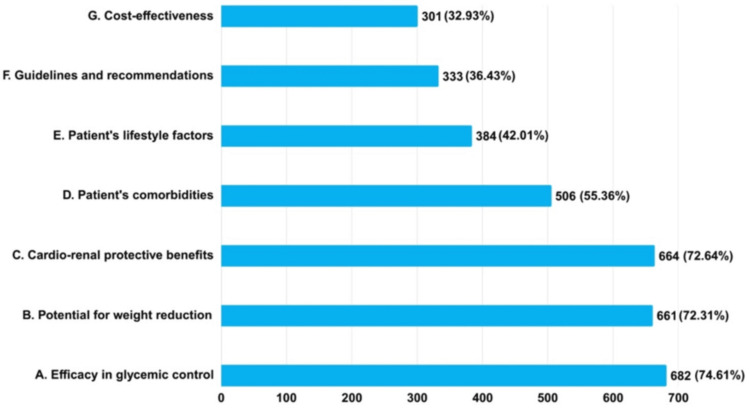
Assessment of clinicians' attitudes regarding primary factors influencing their decision to prescribe combination therapy of dapagliflozin and metformin for T2DM patients (N = 914) T2DM: type 2 diabetes mellitus

The findings showed that 682 (74.61%) clinicians considered efficacy in glycemic control as the primary factor influencing their decision to prescribe combination therapy of dapagliflozin and metformin (Dapa-Met) for T2DM patients, whereas 661 (72.31%) considered the potential for weight reduction. Consequently, 664 (72.64%) clinicians considered the cardiorenal protective effects crucial. A total of 506 (55.36%) clinicians considered patient comorbidities, 384 (42.01%) emphasized lifestyle factors, 333 (36.43%) valued guideline recommendations, and 301 (32.93%) considered cost-effectiveness (Figure 2).

Practice patterns in managing obesity in T2DM

**Figure 3:**
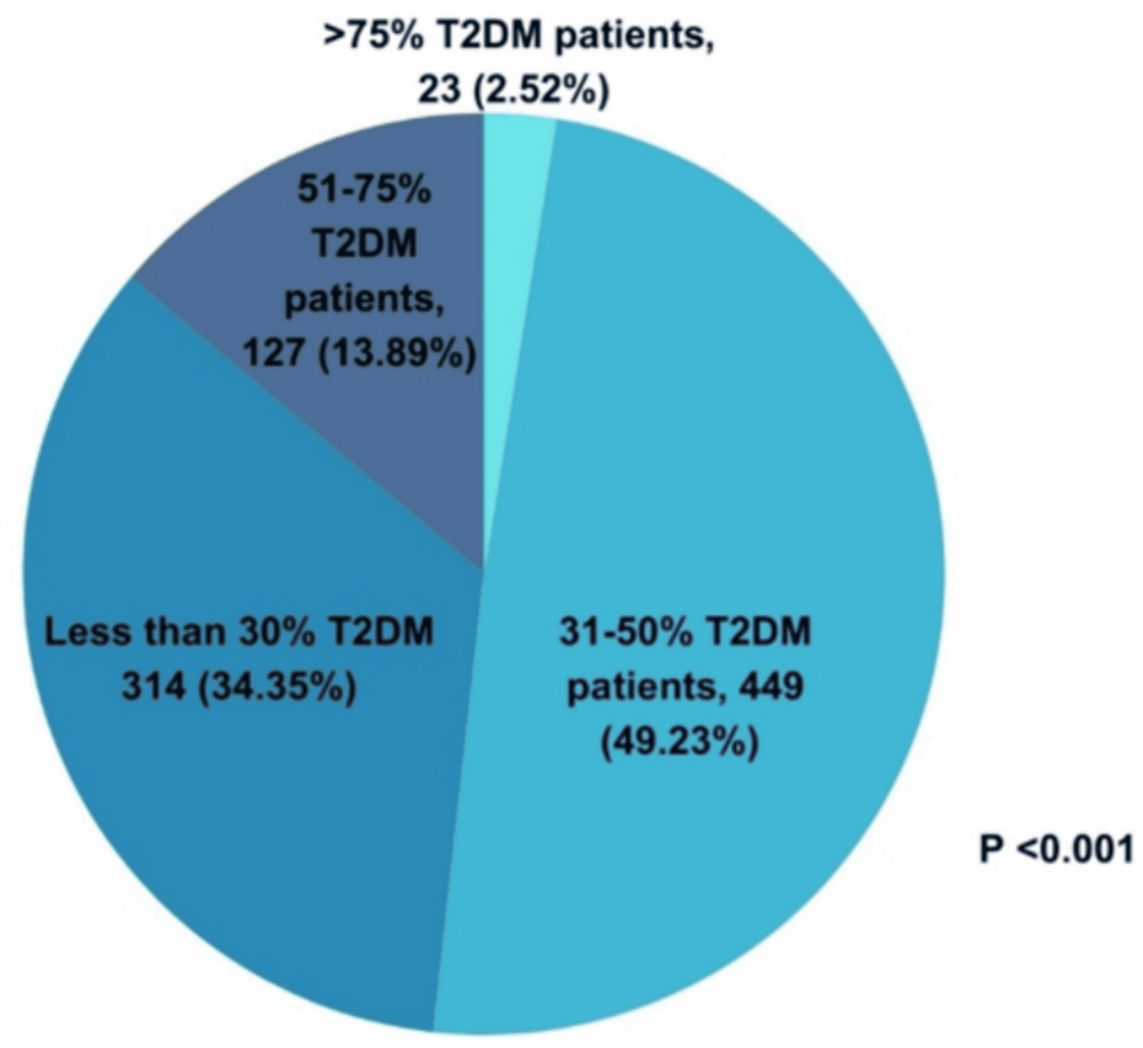
Clinicians’ practice assessment on the number of overweight T2DM patients encountered in their daily clinical practice (N = 914) T2DM: type 2 diabetes mellitus

On a day-to-day basis, 450 (49.23%) clinicians encountered 31%-50% overweight T2DM patients, 314 (34.35%) encountered <30%, and 23 (2.52%) encountered >75% overweight T2DM patients (Figure 3).

**Figure 4:**
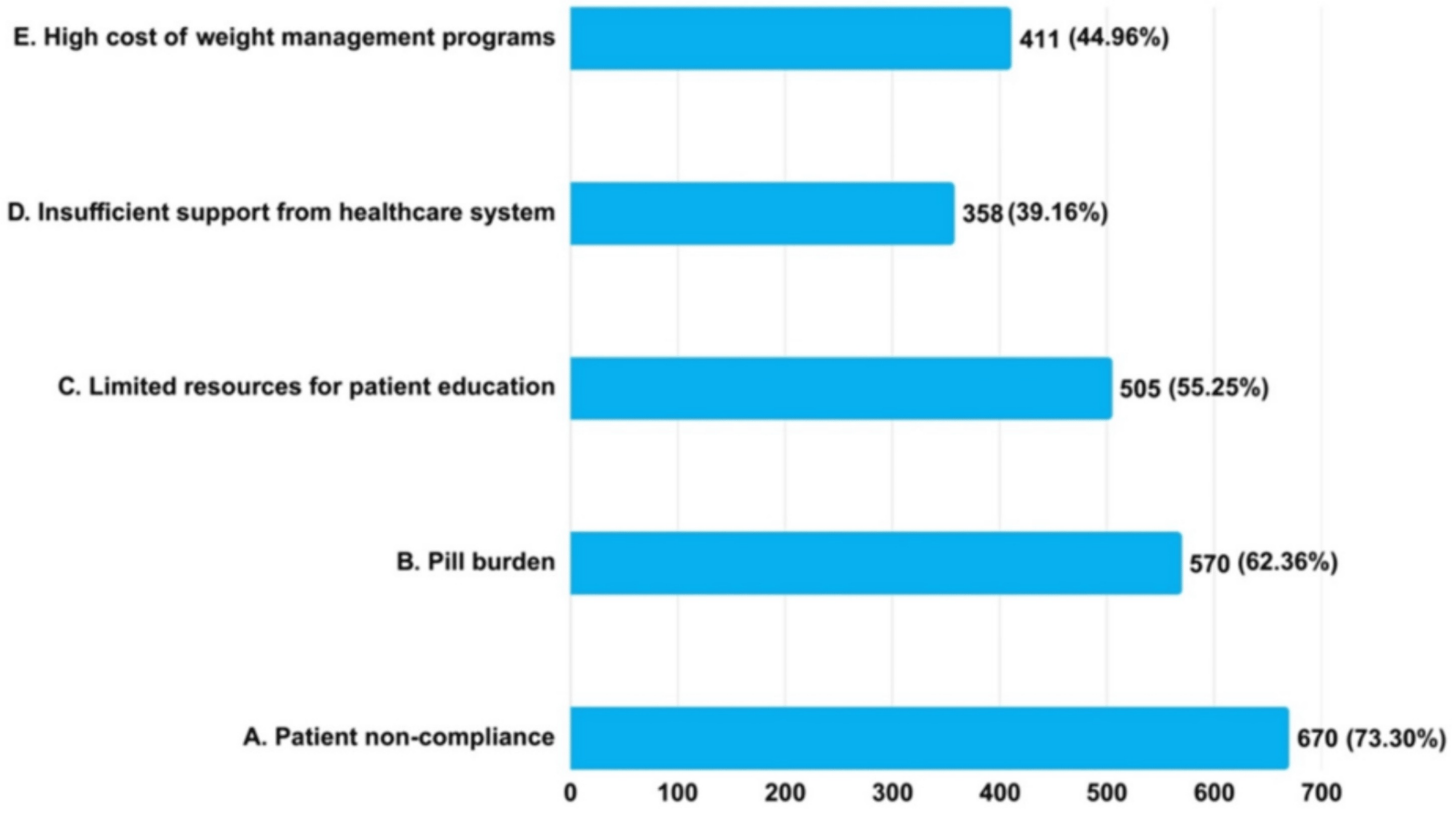
Clinicians’ practice assessment regarding barriers encountered in managing obesity in T2DM patients (N = 914) T2DM: type 2 diabetes mellitus

Most clinicians considered patient noncompliance to be a significant barrier, 670 (73.30%). Similarly, 570 (62.36%) clinicians considered the pill burden, 505 (55.25%) considered limited patient education resources, and 358 (39.6%) noted insufficient support from the healthcare system. Nearly half of those surveyed, 411 (44.96%), viewed the expensive nature of weight management programs as a significant impediment to effectively addressing obesity in T2DM patients (Figure 4).

**Figure 5:**
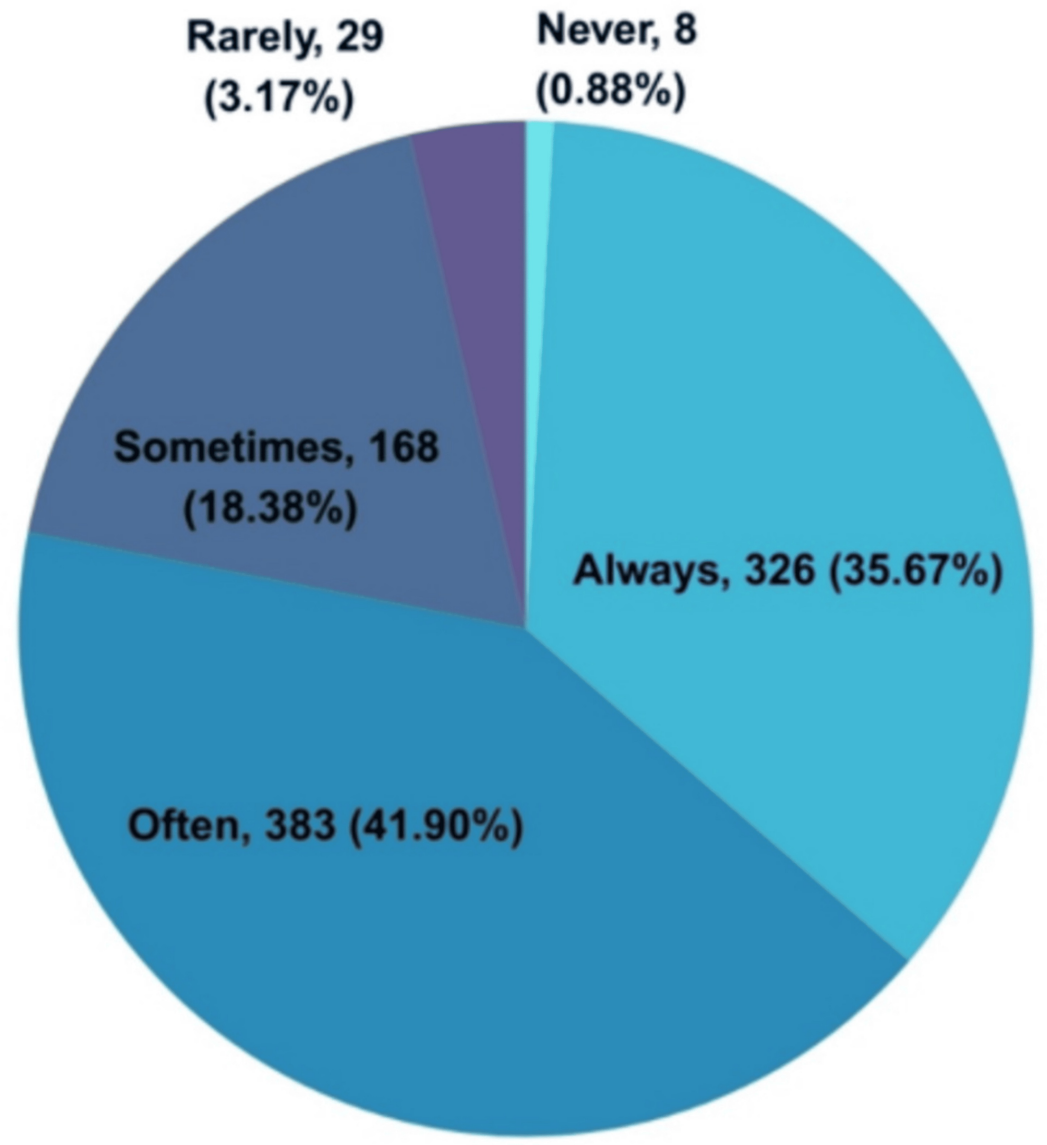
Clinicians’ practice assessment on the frequency of prescribing weight management medications to their T2DM patients (N = 914) T2DM: type 2 diabetes mellitus; N: total number of participants

Of the 914, 326 (35.67%) clinicians consistently prescribed weight management medications to their T2DM patients, 383 (41.9%) prescribed them often, and 168 (18.38%) prescribed them sometimes. Only 8 (0.88%) clinicians responded that they had never prescribed weight management medications to their T2DM patients (Figure 5).

**Figure 6:**
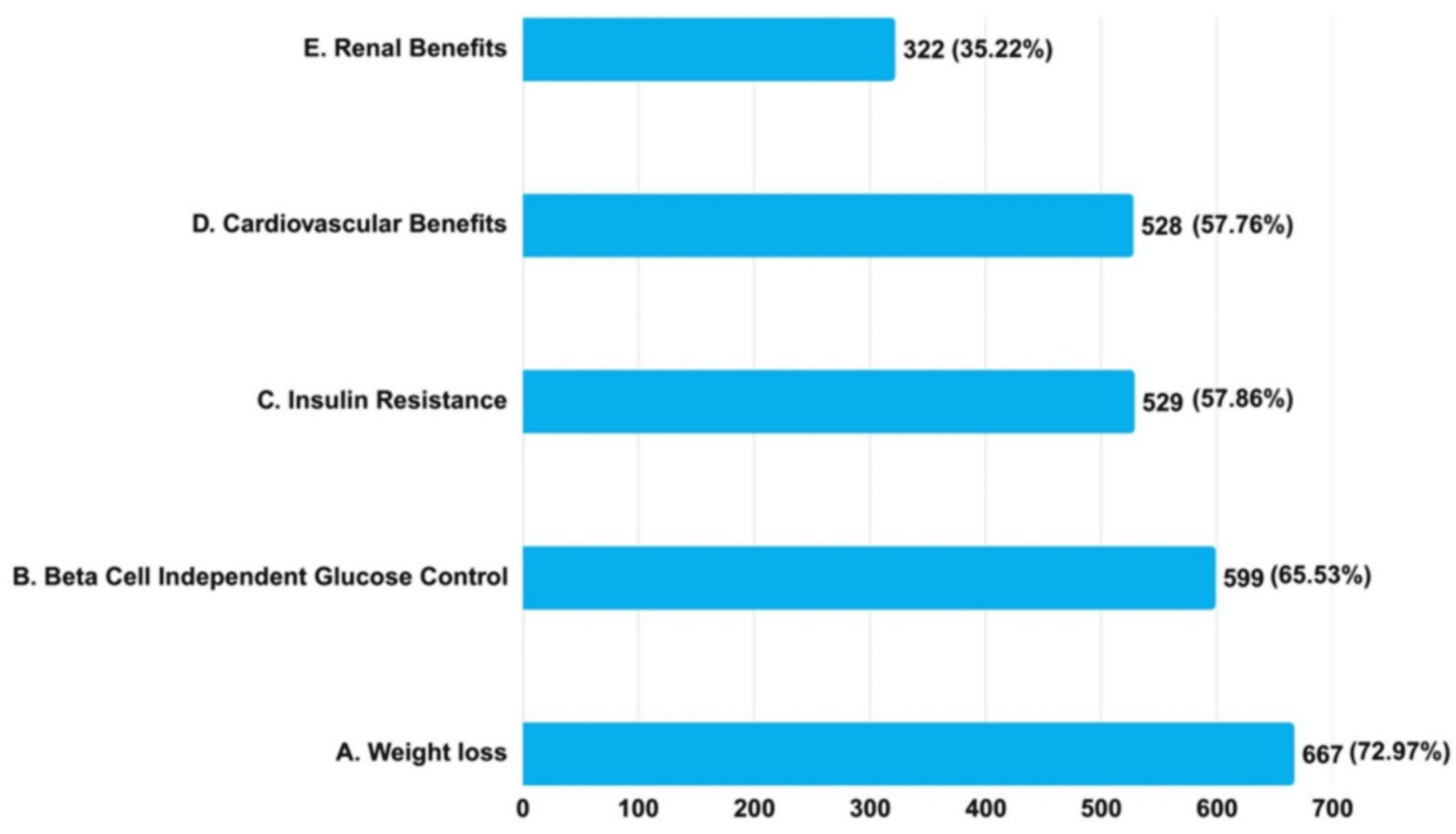
Clinicians' practice assessment on reasons for considering SGLT2i/metformin combination as a first choice in overweight T2DM patients (N = 914) SGLT2i: sodium-glucose cotransporter-2 inhibitors; T2DM: type 2 diabetes mellitus

Among the 914 clinicians, 667 (72.97%) prioritized weight reduction, 599 (65.53%) favored beta-cell-independent glucose regulation, 529 (57.86%) focused on managing IR, and 528 (57.76%) emphasized CV benefits when prescribing the SGLT2i/metformin combination to overweight T2DM patients (Figure 6).

**Figure 7:**
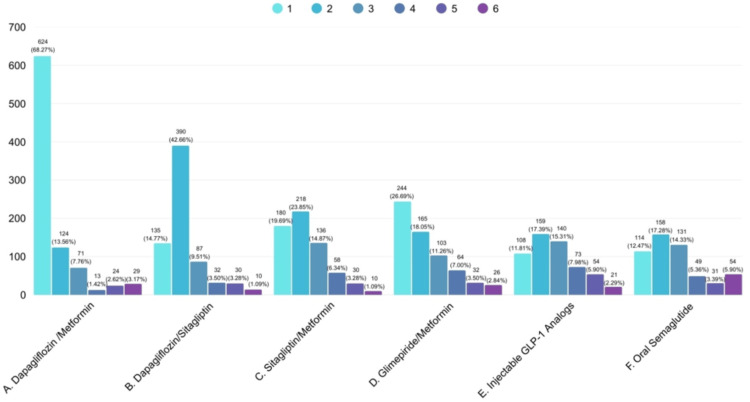
Clinicians’ practice assessment on preferred initial combination of glucose-lowering drugs in overweight T2DM patients (N = 914) T2DM: type 2 diabetes mellitus; GLP-1: glucagon-like peptide-1; 1: first preference; 2: second preference; 3: third preference; 4: fourth preference; 5: fifth preference; 6: sixth preference

Dapagliflozin+metformin was the first preferred initial combination of glucose-lowering drugs in overweight T2DM patients by 624 (68.27%) clinicians, followed by 390 (42.66%) who preferred dapagliflozin+sitagliptin. Oral semaglutide was the sixth or last preferred medication by 54 (7.00%) clinicians (Figure 7).

Table 4 illustrates the assessment of clinicians' practices regarding the most frequently prescribed medications for obesity management in T2DM patients across various scenarios. Of the 914 clinicians, 546 responded to question 13 in the survey. The preferred initial combination therapy varies significantly, depending on the clinical situation. Among newly diagnosed overweight T2DM patients, 414 (75.82%) clinicians favored dapagliflozin+metformin FDC, 95 (17.40%) chose dapagliflozin+sitagliptin FDC, and only 37 (6.78%) selected dapagliflozin+metformin+sitagliptin FDC. Among overweight patients with uncontrolled T2DM and HbA1c >9%, 180 (32.97%) clinicians preferred dapagliflozin+metformin+sitagliptin FDC, whereas 295 (54.21%) opted for dapagliflozin+sitagliptin FDC. For long-standing T2DM patients on insulin, 171 (31.32%) clinicians chose dapagliflozin+metformin+sitagliptin FDC, while 262 (46.15%) favored dapagliflozin+sitagliptin FDC.

**Table 4:** Clinicians' practice assessment regarding most commonly prescribed medication for managing obesity in T2DM patients (N = 546) ASCVD: atherosclerotic cardiovascular disease; FDC: fixed-dose combination; CI: confidence interval; T2DM: type 2 diabetes mellitus; LVH: left ventricular hypertrophy; LV: left ventricle; n: number of participants; N: total participants; df: degree of freedom, t-test ^*^One-sample t-test

Select your first choice for each of these scenarios	Dapagliflozin+metformin FDC, n (%)	Dapagliflozin+sitagliptin FDC, n (%)	Dapagliflozin+metformin+sitagliptin FDC, n (%)	p value	95% CI	t-statistics^*^	df
Newly diagnosed overweight T2DM	414 (75.82%)	95 (17.40%)	37 (6.78%)	<0.001	1.21-1.28	51.730	545
Overweight T2DM with hypertension	268 (49.08%)	206 (37.73%)	72 (13.19%)	<0.001	2.37-2.49	54.515	545
Overweight uncontrolled T2DM with HbA1c >7.5%	209 (38.28%)	264 (48.35%)	73 (13.37%)	<0.001	2.32-2.44	60.635	545
Overweight uncontrolled T2DM with HbA1c >9%	70 (12.82%)	295 (54.21%)	180 (32.97%)	<0.001	2.07-2.14	79.560	545
Young T2DM uncontrolled on metformin	289 (52.93%)	192 (35.16%)	65 (11.90%)	<0.001	1.54-1.64	53.566	545
T2DM with ASCVD	115 (21.06%)	346 (63.37%)	85 (15.57%)	<0.001	1.86-1.95	75.337	545
T2DM with albuminuria	160 (29.30%)	270 (47.62%)	126 (23.08%)	<0.001	1.87-1.98	62.736	545
T2DM with LVH/LV dysfunction	127 (23.26%)	297 (54.40%)	122 (22.34%)	<0.001	1.90-2.01	68.829	545
Long-standing T2DM on insulin	123 (22.53%)	262 (46.15%)	171 (31.32%)	<0.001	2.01-2.12	66.907	545
T2DM with heart failure	121 (22.16%)	321 (58.79%)	104 (19.05%)	<0.001	1.90-2.00	71.685	545

Appendices 2, 3 demonstrate variations in the KAP of Indian clinics in managing obesity in T2DM patients and the prescribing practices of dapagliflozin+metformin and dapagliflozin+sitagliptin FDCs in managing obesity in T2DM patients. Initial drug of choice preference for dapagliflozin+metformin and dapagliflozin+sitagliptin FDCs among varied case scenarios across specialties and regions. The trends were similar across all specialties and regions (p < 0.001), indicating a consistent pattern of views among all clinicians.

Zone-wise and specialty-wise association

Clinicians across all zones, including the north, south, west, east, and central regions, preferred dapagliflozin+metformin FDC. Similar trends were observed among clinicians from various specialties (Appendices 4, 5). All the questions analyzed zone-wise and specialty-wise were statistically significant (p < 0.001).

## Discussion

This cross-sectional survey assessed the current understanding, perspectives, and approaches of Indian clinicians in managing obesity in T2DM patients. This study demonstrated reasonable KAP of Indian clinicians adhering to clinical guidelines, acknowledging the critical role of weight control in T2DM treatment, and prescribing FDCs with dapagliflozin for weight loss in T2DM patients. The significant barriers clinicians considered were medication adherence, pill burden, patient education, and the high costs of weight management programs. There was a high preference for interventions, such as dapagliflozin+metformin FDCs, followed by dapagliflozin+sitagliptin FDCs, for obesity management in T2DM patients, overweight T2DM patients with hypertension, uncontrolled T2DM with HbA1c >9%, and T2DM with atherosclerotic cardiovascular disease (ASCVD) and HF.

Indian clinicians’ perspectives regarding obesity management in patients with T2DM are as follows.

1) Weight management should be the primary goal of treatment, in addition to glycemic management, for overweight or obese patients with T2DM.

2) Overweight/obese patients living with T2DM may benefit from 3% to 7% of baseline weight loss.

3) Lifestyle interventions are effective in managing obesity in T2DM patients.

4) Patient nonadherence is the main challenge in managing obesity in T2DM patients, along with medication burden, limited education resources, insufficient healthcare support, and high program costs.

5) For overweight T2DM patients, dapagliflozin+metformin FDC can be the preferred initial glucose-lowering drug combination, followed by dapagliflozin+sitagliptin FDC as the second choice.

6) Dapagliflozin+metformin FDC can be preferred for newly diagnosed overweight T2DM patients and with hypertension.

7) Both dapagliflozin+metformin and dapagliflozin+sitagliptin FDCs can be preferred for overweight, uncontrolled T2DM with HbA1c >9%, T2DM with ASCVD, and T2DM with HF.

These specialist perspectives can inform clinical decision-making regarding the optimal management of this high-risk population. However, limited data exist on Indian clinicians' understanding of and approaches to managing obesity in T2DM patients. Perceptions about prescribing Dapa-Met for obesity management in T2DM remain unclear. Therefore, we compared our survey findings with those of the guidelines, trials, and real-world studies.

Participating clinicians agreed that overweight T2DM patients had more frequent uncontrolled blood glucose levels than normal patients, aligning with the ADA and European Association for the Study of Diabetes (EASD) Consensus Report, which shows that increased impaired glucose tolerance and T2DM correlate with obesity rates [30]. Clinicians in the current survey agreed to recommend weight management as a primary treatment goal alongside glycemic management for overweight T2DM patients and recognized the benefits of 3%-7% baseline weight reduction, aligning with ADA and EASD recommending a 5% target, noting that >10% weight loss early in T2DM increases remission chances [30]. According to the International Diabetes Federation, the global increase in obesity contributes to the increasing prevalence of T2DM, a chronic condition characterized by inadequate insulin production, resulting in hyperglycemia [31]. Obesity and T2DM share both genetic and environmental factors in their pathogenesis, with obesity exacerbating genetic predisposition and environmental influences. The expansion of adipose tissue and nutrient accumulation disrupt metabolic equilibrium through mechanisms such as IR and the microbiome-gut-brain axis, ultimately leading to β-cell loss and elevated blood glucose levels [32]. Consequently, effective weight management in T2DM requires personalized strategies tailored to individual patient needs.

Clinicians in the current survey reported CVDs, hypertension, microvascular complications, dyslipidemia, and CKD as significant complications of obesity in T2DM patients and agreed that lifestyle interventions effectively manage obesity. A task force of experts in cardiology, nephrology, endocrinology, and primary care emphasized that T2DM, obesity, and cardiorenal/metabolic diseases commonly co-occur. Despite new medications, metabolic control remains suboptimal after 20 years. Traditional stepwise treatments often cause therapeutic inertia, increasing morbidity and mortality. Early intensive combinations can prevent disease progression. These recommendations include screening, early lifestyle interventions, and guidelines for combination oral antihyperglycemic agents [33].

Most clinicians in the current survey agreed to prescribe dapagliflozin+metformin FDC based on Indian real-world evidence (ADMIRE Study), showing its preference for weight loss beyond glucose control. International guidelines recommend SGLT2i as a first-line treatment in T2DM patients [19,34]. The use of SGLT2i with metformin is preferred for managing T2DM [34,35]. The ADMIRE study evaluated prescribing patterns for T2DM patients initiated on Dapa-Met FDC. Data from 485 T2DM patients (mean age: 59.7 ± 9.8 years) with a mean diabetes duration of 6.9 ± 4.7 years were analyzed. For Dapa-Met FDC, 10 and 500 mg were most commonly preferred. The FDC was favored for its benefits in weight loss (77.1%) and reduced CV events and hospitalizations (46.8%) [35]. In the current survey, clinicians prioritized glycemic control, weight reduction, cardiorenal protection, patient comorbidities, lifestyle factors, guidelines, and cost-effectiveness when prescribing FDC. This aligns with Phadke et al.’s study of 481 T2DM patients across 49 clinical units, where Dapa-Met FDC showed good efficacy, tolerability, and safety. This combination was preferred for weight loss promotion and CV-renal benefits, with its safety contributing to improved treatment compliance. The FDC proved effective and well-tolerated as an initial therapy for T2DM in Indian populations [36].

In the current survey, clinicians prioritized weight reduction, beta-cell-independent glucose regulation, IR management, and CV benefits when prescribing SGLT2i/metformin to overweight T2DM patients. This aligns with the review by Singh et al., highlighting SGLT2i's benefits in Indian patients with T2DM through abdominal fat loss. SGLT2i's improved β-cell function and reduced IR are particularly useful in Indian patients with T2DM. In T2DM patients with inadequate glycemic control on metformin monotherapy, SGLT2i are preferred as second-line agents. SGLT2i reduce HbA1C, FPG, body weight, and BP in patients with T2DM. As a second-line therapy, SGLT2i improve glycemic control and body weight while providing CV and renoprotective benefits [37]. In the current survey, most clinicians preferred dapagliflozin+metformin as the initial combination for overweight T2DM patients. This aligns with Sethi et al.'s multicenter study, showing dapagliflozin with metformin significantly reducing HbA1c levels (-1.1 ± 1.44%; p < 0.05 for HbA1c subgroup ≥7.5%; -1.6 ± 1.41%; p < 0.05 for HbA1c subgroup ≥8%) and body weight (-1.4 ± 3.31 kg; p < 0.05 for HbA1c subgroup ≥7.5%; -1.5 ± 3.22 kg; p < 0.05 for HbA1c subgroup ≥8%) at visit 2. A significant BMI change was noted for the HbA1c subgroup ≥7.5% (-1.0 ± 8.38 kg/m²) [17].

The FDA has approved canagliflozin, dapagliflozin, and empagliflozin as SGLT-2i [38]. These inhibitors show favorable effects on body weight, BP, dyslipidemia, and fatty liver disease, while lowering the risk of hypoglycemia with positive CV and renal safety results. SGLT-2i serve as second-line antidiabetic drugs when first-line medications fail, although they may be used alone [39]. Varshney and Rawat reviewed the safety profiles of SGLT-2i in type 2 diabetes patients. The review highlighted the findings by Viswanathan and Singh that Indian patients starting dapagliflozin had a mean HbA1c of 9.11 ± 1.44%, with 56.3% between 8% and 10%, while empagliflozin patients showed a mean HbA1c of 7.92 ± 0.7018 [16,39]. Dapagliflozin patients lost 1.14 (2.21) kg at three months and 1.86 (3.04) kg at six months (SD). Those with BMI >30 lost, mean (SD): 1.60 (2.50) kg, at three months and 2.56 (3.50) kg at six months. Empagliflozin treatment (10 or 25 mg) for 76 weeks decreased the body weight by 1.41 and 1.50 kg [39,40].

In the present survey, clinicians preferred injectable GLP-1 analogs as their third, fourth, and fifth preferences. A minority of clinicians selected oral semaglutide for their sixth preference. The diabetes management landscape has evolved with new therapeutic options, particularly for GLP-1 RAs. Palanca et al. mentioned that these therapies improve glycemic control, weight management, and CV outcomes, empowering the modern T2DM armamentarium. GLP‐1RAs enhance glycemic control and promote weight loss, with a low risk of hypoglycemia. Zarrinkamar et al. and Ciardullo et al. showed higher medication adherence among GLP‐1RA users (75.2%) than among nonusers (71.5%), with reduced hospitalizations among adherent patients. These outcomes demonstrate GLP-1RAs' importance in adult obesity T2DM management [41-43]. In the current survey, most clinicians reported patient noncompliance as a significant barrier, followed by the pill burden, limited patient education resources, insufficient support from the healthcare system, and the expensive nature of weight management programs as significant barriers to effectively addressing obesity in T2DM patients. Das et al., in their study on the healthcare delivery model in India with respect to diabetes care, emphasize that factors such as lack of awareness, psychological barriers, individual health issues, inadequate healthcare facilities, limited access, high costs, nonadherence to treatment, deficiencies in medical infrastructure, insufficient supplies, economic disparities, and challenges in accessing quality healthcare facilities hamper effective management of T2DM [44].

Lifestyle modifications, including dietary changes and physical activity, are established treatments for T2DM and obesity, and are recommended as first-line strategies. Clinicians should inform overweight/obese T2DM patients that fat accumulation worsens T2DM complications, CVDs, mortality, and quality of life. Assessing patients' preparedness for weight loss modification is crucial before establishing individualized objectives through shared decision-making. Healthcare providers should focus on prescribing drugs that offer glycemic control and positive weight outcomes in T2DM management to manage HbA1c levels and body weight simultaneously. Agents associated with weight loss include SGLT2i (dapagliflozin), GLP-1 RAs, dual gastric inhibitory polypeptide (GIP) and GLP-1 RAs (tripeptides), metformin, and amylin mimetics. Overweight/obese T2DM patients prefer medicines with positive weight impact, such as GLP-1 RAs, dual GIP and GLP-1 RAs (tripeptides), dapagliflozin, metformin, and sitagliptin FDCs. Regular assessments of weight management and annual monitoring of obesity-related anthropometric data are recommended [30,45-48].

Strengths and limitations

The primary strength of this survey was the participation of 914 clinicians from diverse regions and healthcare settings across India, providing a representative understanding of current practices. This survey addressed obesity management in T2DM patients, as obesity significantly affects T2DM progression and outcome. The results identified knowledge gaps and areas for practice improvement, potentially informing educational programs or guideline updates. The distribution of the survey across various regions and specialties has significantly enhanced its comprehensive coverage. However, the survey was not without its limitations. The cross-sectional design restricted the determination of cause-and-effect relationships across periods. Self-reported data may introduce information, recall, and social desirability biases, with respondents potentially overstating guideline adherence. Factors such as practice type, patient demographics, and clinician experience were not controlled. Convenience sampling restricts the generalizability of the findings because the selected respondents may not adequately represent the comprehensive diversity of India's healthcare population. While the survey was anonymous and voluntary, obtaining institutional ethical clearance would strengthen its credibility. Because this was a cross-sectional survey, it lacked a control group and patient-reported outcome data. To further support the survey findings, we recommend phase IV or real-world studies to assess the long-term benefits of Dapa-Met FDCs on sustained weight loss in T2DM patients in Indian clinical settings.

## Conclusions

This PAN India survey provides insights into Indian clinicians' understanding of obesity management in T2DM patients and their views on combination treatments with dapagliflozin in Indian clinical practice. These findings show that clinicians recognize the importance of glycemic control, weight management, and cardiorenal protection, with variations in their implementation. The survey revealed that clinicians prefer dapagliflozin+metformin FDCs, followed by dapagliflozin+sitagliptin FDCs, for newly diagnosed overweight T2DM patients, particularly those with hypertension and HF, owing to their efficacy in glycemic control, weight reduction, and cardiorenal benefits. However, patient noncompliance, pill burden, limited resources, insufficient healthcare support, and high costs are significant barriers to managing obesity in T2DM patients. Given the "Twindemic" of obesity and T2DM in India, dapagliflozin-based combination therapies can be considered a potential therapeutic option for managing obesity in T2DM patients.

## Appendices

Appendix 1

**Table 5:** T2DM and obesity: HCP perception KAP survey T2DM: type 2 diabetes mellitus; ADMIRE: Assessing Dapagliflozin And Metformin As An Initial Regimen In Diabetes Mellitus For Enhanced Glucose Control; FDC: fixed-dose combinations; SGLT2i: sodium-glucose cotransporter-2 inhibitors; GLP-1: glucagon-like peptide-1; ASCVD: atherosclerotic cardiovascular disease; LVH/LV: left ventricular hypertrophy/left ventricle; HCP: healthcare professional; KAP: knowledge, attitudes, and practices

Knowledge						
1. Overweight T2DM patients have uncontrolled blood sugar levels more often than normal-weight T2DM patients. A. Strongly agree. B. Agree. C. Undecided. D. Disagree. E. Strongly disagree.						
2. Guidelines recommend that among overweight or obese people living with T2DM, weight management should represent a primary goal of treatment along with glycemic management. A. Strongly agree. B. Agree. C. Undecided. D. Disagree. E. Strongly disagree.						
3. Guidelines recommend that overweight or obese people living with T2DM may benefit from 3% to 7% of baseline weight loss. A. Strongly agree. B. Agree. C. Undecided. D. Disagree. E. Strongly disagree.						
4. Which of the following are major complications associated with obesity in type 2 diabetes patients? (Select all that apply) A. Cardiovascular diseases. B. Hypertension. C. Dyslipidemia. D. Microvascular complications. E. Kidney disease. Other:						
Attitude						
5. What are the primary factors influencing your decision to prescribe the combination therapy of dapagliflozin and metformin for people with T2DM? (Select all that apply) A. Efficacy in glycemic control. B. Potential for weight reduction. C. Cardiorenal protective benefits. D. Patient's comorbidities. E. Patient's lifestyle factors. F. Guidelines and recommendations. G. Cost-effectiveness. 6. Do you believe that lifestyle interventions are effective in managing obesity in type 2 diabetes patients? A. Strongly agree. B. Agree. C. Neutral. D. Disagree. E. Strongly disagree.						
6. Do you believe that lifestyle interventions are effective in managing obesity in type 2 diabetes patients? A. Strongly agree. B. Agree. C. Neutral. D. Disagree. E. Strongly disagree.						
7. Most recent Indian real-world evidence (ADMIRE study) indicates that Dapagliflozin Metformin FDC is mainly preferred for weight loss beyond glucose control. What are your views? A. Strongly agree. B. Agree. C. Undecided. D. Disagree. E. Strongly disagree.						
Practices						
8. How many overweight T2DM patients do you encounter in your daily clinical practice? A. Less than 30% T2DM patients. B. 31-50% T2DM patients. C. 51-75% T2DM patients. D. >75% T2DM patients						
9. What are the barriers you face in managing obesity in type 2 diabetes patients? (Select all that apply) A. Patient noncompliance. B. Pill burden. C. Limited resources for patient education. D. Insufficient support from healthcare system. E. High cost of weight management programs. Other:						
10. How often do you prescribe medications specifically for weight management in your type 2 diabetes patients? A. Always. B. Often. C. Sometimes. D. Rarely. E. Never.						
11. Among overweight people living with T2DM, SGLT2i/metformin combination could be the first consideration, unless contraindicated due to the following reasons: A. Weight loss. B. Beta-cell independent glucose control. C. Insulin resistance. D. Cardiovascular benefits. E. Renal benefits.						
12. What is your preferred initial combination of glucose-lowering drugs among overweight people living with T2DM? (Please rank in order of preference)						
A. Dapagliflozin/metformin. B. Dapagliflozin/sitagliptin. C. Sitagliptin/metformin. D. Glimepiride/metformin. E. Injectable GLP-1 analogs. F. Oral semaglutide	1	2	3	4	5	6
	-	-	-	-	-	-
13. Select your first choice for each of these scenarios						
-		Dapagliflozin+Metformin FDC		Dapagliflozin+Sitagliptin FDC		Dapagliflozin+Sitagliptin+Metformin FDC
Newly diagnosed overweight T2DM, Overweight T2DM with hypertension, Overweight uncontrolled T2DM with HbA1c >7.5%, Overweight uncontrolled T2DM with HbA1c >9%, Young T2DM uncontrolled on metformin T2DM with ASCVD, T2DM with albuminuria T2DM with LVH/LV dysfunction, and Long-standing T2DM on insulin T2DM with heart failure		-		-		-

Appendix 2

**Table 6:** Variations in clinicians' knowledge, practice, and attitude assessment regarding managing obesity in T2DM patients (N = 914) FDC: fixed-dose combination; ADMIRE: Assessing Dapagliflozin And Metformin As An Initial Regimen In Diabetes Mellitus For Enhanced Glucose Control; T2DM: type 2 diabetes mellitus; CI: confidence interval

Question	Region-wise		Specialty-wise	
	p value	95% CI	p value	95% CI
Overweight T2DM patients have uncontrolled blood sugar levels more often than normal-weight T2DM patients	<0.001	-1.784 to 1.590	<0.001	-0.863 to 0.699
Guidelines recommend that among overweight or obese people living with T2DM, weight management should represent a primary goal of treatment along with glycemic management	<0.001	-1.754 to -1.564	<0.001	-0.833 to -0.673
Guidelines recommend that overweight or obese people living with T2DM may benefit from 3% to 7% of baseline weight loss	<0.001	-1.640 to -1.445	<0.001	-0.718 to -0.055
Do you believe that lifestyle interventions are effective in managing obesity in T2DM patients?	<0.001	-1.694 to -1.501	<0.001	-0.773 to -.601
Most recent Indian real-world evidence (ADMIRE Study) indicates that dapagliflozin metformin FDC is mainly preferred for weight loss beyond glucose control. What are your views?	<0.001	1.443 to 1.638	<0.001	0.556 to 0.714
How many overweight T2DM patients do you encounter in your daily clinical practice?	<0.001	-0.498 to -0.294	<0.001	0.428 to 0.592
How often do you prescribe medications specifically for weight management in your T2DM patients?	<0.001	-0.433 to -0.217	<0.001	0.493 to 0.699

Appendix 3

**Table 7:** Variations in clinicians' practice assessment regarding most commonly prescribed medication for managing obesity in T2DM patients (N = 546) T2DM: type 2 diabetes mellitus; LVH: left ventricular failure; LV: left ventricle; ASCVD: atherosclerotic cardiovascular disease; CI: confidence interval; HbA1c: hemoglobin A1c

Question (select your first choice for each of these scenarios)	Region-wise		Specialty-wise	
	p value	95% CI	p value	95% CI
a. Newly diagnosed overweight T2DM	<0.001	1.882 to 2.079	<0.001	1.015 to 1.171
b. Overweight T2DM with hypertension	<0.001	-1.709 to -1.486	<0.001	-0.837 to -0.662
c. Overweight uncontrolled T2DM with HbA1c >7.5%	<0.001	-1.607 to -1.383	<0.001	-0.723 to -0.542
d. Overweight uncontrolled T2DM with HbA1c >9%	<0.001	-1.210 to -0.983	<0.001	-0.329 to -1.48
e. Young T2DM uncontrolled on metformin	<0.001	-1.720 to -1.488	<0.001	-0.877 to -0.682
f. T2DM with ASCVD	<0.001	-1.436 to -1.207	<0.001	-0.548 to -0.363
g. T2DM with albuminuria	<0.001	-1.407 to -1.163	<0.001	-0.564 to -0.357
h. T2DM with LVH/LV dysfunction	<0.001	-1.387 to -1.149	<0.001	-0.532 to -0.330
i. Long-standing T2DM on insulin	<0.001	-1.281 to -1.041	<0.001	-0.427 to -0.227
j. T2DM with heart failure	<0.001	-1.408 to -1.179	<0.001	-0.524 to -0.324

Appendix 4

**Table 8:** Region-wise clinicians’ practice assessment regarding most commonly prescribed medication for managing obesity in T2DM patients (N = 546) FDC: fixed-dose combination; T2DM: type 2 diabetes mellitus; LVH: left ventricular hypertrophy; NR: no response; n: number of participants; N: total participants; ASCVD: atherosclerotic cardiovascular disease

Select your first choice for each of these scenarios	Region-wise														
	Dapagliflozin + metformin FDC, n (%)					Dapagliflozin + sitagliptin FDC, n (%)					Dapagliflozin + metformin + sitagliptin FDC, n (%)				
	North	South	East	West	Central	North	South	East	West	Central	North	South	East	West	Central
Newly diagnosed overweight T2DM	69 (12.64%)	118 (21.79%)	78 (14.29%)	94 (17.22%)	53 (9.89%)	6 (1.10%)	19 (3.48%)	27 (4.95%)	22 (4.03%)	21 (3.85%)	3 (0.55%)	12 (2.38%)	NR	6 (1.28%)	13 (2.56%)
Overweight T2DM with hypertension	40 (7.33%)	67 (12.45%)	53 (9.89%)	64 (11.90%)	41 (7.51%)	32 (6.04%)	57 (10.62%)	28 (5.31%)	47 (8.79%)	38 (6.96%)	5 (0.92%)	25 (4.58%)	22 (4.03%)	9 (1.83%)	9 (1.83%)
Overweight uncontrolled T2DM with HbA1c >7.5%	29 (5.49%)	64 (11.90%)	57 (10.62%)	28 (5.13%)	28 (5.13%)	40 (7.33%)	67 (12.45%)	40 (7.33%)	79 (14.65%)	35 (6.59%)	8 (1.47%)	30 (3.30%)	11 (1.28%)	25 (2.75%)	41 (4.58%)
Overweight uncontrolled T2DM with HbA1c >9%	8 (1.47%)	19 (3.66%)	12 (2.20%)	12 (2.20%)	18 (3.30%)	41 (7.69%)	88 (16.30%)	54 (10.07%)	71 (13.19%)	38 (6.96%)	28 (5.13%)	41 (7.69%)	38 (6.96%)	39 (7.14%)	32 (6.04%)
Young T2DM uncontrolled on metformin	34 (6.41%)	92 (16.85%)	63 (11.54%)	56 (10.26%)	43 (7.88%)	31 (5.86%)	38 (6.96%)	29 (5.49%)	57 (10.44%)	34 (6.41%)	10 (2.01%)	21 (3.85%)	12 (2.20%)	9 (1.83%)	12 (2.01%)
T2DM with ASCVD	17 (3.11%)	31 (5.68%)	15 (2.93%)	28 (5.13%)	22 (4.21%)	44 (8.06%)	86 (15.93%)	82 (15.02%)	82 (15.20%)	50 (9.16%)	16 (3.11%)	32 (6.04%)	6 (1.28%)	12 (2.20%)	16 (2.93%)
T2DM with albuminuria	17 (3.11%)	44 (8.06%)	38 (6.96%)	33 (6.04%)	28 (5.13%)	38 (6.96%)	66 (12.09%)	39 (7.14%)	71 (13.00%)	46 (8.42%)	23 (4.21%)	41 (7.51%)	28 (5.13%)	19 (3.48%)	15 (2.75%)
T2DM with LVH/LV dysfunction	16 (2.93%)	27 (4.95%)	29 (5.31%)	32 (5.86%)	32 (5.86%)	45 (8.24%)	99 (18.13%)	44 (8.06%)	64 (11.72%)	38 (6.96%)	36 (6.59%)	25 (4.58%)	19 (3.48%)	19 (3.48%)	21 (3.85%)
Long-standing T2DM on insulin	32 (6.04%)	50 (9.16%)	41 (7.51%)	28 (5.13%)	19 (3.48%)	34 (6.23%)	71 (13.00%)	70 (7.14%)	66 (12.27%)	41 (7.51%)	10 (2.01%)	29 (5.49%)	25 (4.58%)	28 (5.13%)	28 (5.31%)
T2DM with heart failure	21 (3.85%)	18 (5.31%)	20 (3.66%)	22 (4.03%)	18 (5.31%)	35 (6.41%)	91 (16.67%)	73 (13.55%)	76 (14.10%)	44 (8.06%)	22 (4.03%)	31 (5.68%)	10 (2.01%)	24 (4.40%)	16 (2.93%)

Appendix 5

**Table 9:** Speciality-wise clinicians' practice assessment regarding the most commonly prescribed medication for managing obesity in T2DM patients (N = 546) CP: consultant physician; GP: general practitioner; FDC: fixed-dose combination; T2DM: type 2 diabetes mellitus; LVH: left ventricular hypertrophy; NR: no response; n: number of participants; N: total participants; ASCVD: atherosclerotic cardiovascular disease

Select your first choice for each of these scenarios	Speciality Wise																	
	Dapagliflozin+ Metformin FDC n (%)						Dapagliflozin + sitagliptin FDC n (%)						Dapagliflozin + sitagliptin FDC n (%)					
	Cardio	CP	Diabeto	Endo	GP	Others	Cardio	CP	Diabeto	Endo	GP	Others	Cardio	CP	Diabeto	Endo	GP	Others
Newly Diagnosed Overweight T2DM	61 (11.17%)	227 (41.58%)	73 (13.37%)	22 (4.21%)	25 (4.58%)	5 (0.92%)	15 (2.75%)	45 (8.24%)	24 (4.40%)	4 (0.73%)	6 (1.10%)	1 (0.18%)	9 (1.83%)	15 (2.75%)	4 (0.73%)	5 (0.92%)	3 (0.55%)	NR
Overweight T2DM with Hypertension	40 (7.33%)	150 (27.47%)	43 (7.88%)	16 (2.93%)	15 (2.75%)	4 (0.73%)	33 (6.04%)	108 (19.78%)	39 (7.14%)	12 (2.20%)	13 (2.38%)	1 (0.18%)	13 (2.38%)	29 (5.31%)	19 (3.48%)	4 (0.73%)	6 (1.10%)	1 (0.18%)
Overweight Uncontrolled T2DM with HbA1c >7.5%	53 (5.86%)	100 (18.32%)	46 (8.42%)	15 (2.75%)	11 (2.01%)	5 (0.92%)	40 (7.33%)	158 (28.94%)	38 (6.96%)	12 (2.20%)	15 (2.75%)	1 (0.18%)	14 (2.56%)	29 (5.31%)	17 (3.11%)	5 (0.92%)	8 (1.47%)	NR
Overweight Uncontrolled T2DM with HbA1c >9%	28 (5.13%)	94 (17.22%)	30 (5.49%)	13 (2.38%)	13 (2.38%)	2 (0.37%)	52 (9.52%)	153 (28.02%)	57 (10.44%)	15 (2.75%)	19 (3.48%)	NR	6 (1.10%)	40 (7.33%)	14 (2.56%)	3 (0.73%)	2 (0.37%)	3 (0.73%)
Young T2DM Uncontroll ed on Metformin	75 (8.24%)	147 (26.92%)	55 (10.07%)	17 (3.11%)	20 (3.66%)	5 (0.92%)	27 (4.95%)	111 (20.33%)	32 (5.86%)	11 (2.01%)	11 (2.01%)	NR	14 (2.56%)	48 (5.31%)	14 (2.56%)	3 (0.73%)	3 (0.55%)	1 (0.18%)
T2DM with ASCVD	19 (3.48%)	57 (10.44%)	19 (3.48%)	7 (1.28%)	9 (1.65%)	4 (0.73%)	55 (10.26%)	190 (34.98%)	60 (10.99%)	19 (3.48%)	18 (3.30%)	2 (0.37%)	18 (2.01%)	39 (7.14%)	22 (4.03%)	6 (1.10%)	7 (1.28%)	NR
T2DM with Albuminuria	26 (4.58%)	78 (14.29%)	28 (5.13%)	12 (2.20%)	13 (2.38%)	4 (0.73%)	37 (6.78%)	154 (28. 21%)	46 (8.42%)	5 (0.92%)	17 (3.11%)	4 (0.88%)	24 (4.40%)	55 (10.07%)	27 (4.95%)	25 (2.75%)	3 (0.73%)	1 (0.18%)
T2DM with LVH / LV Dysfunction	19 (3.48%)	66 (12.27%)	21 (3.85%)	8 (1.47%)	8 (1.47%)	4 (0.73%)	46 (8.42%)	153 (28.02%)	63 (11.54%)	14 (2.56%)	19 (3.48%)	2 (0.37%)	21 (3.85%)	67 (12.27%)	17 (3.11%)	10 (1.83%)	7 (1.28%)	NR
Long- Standing T2DM on Insulin	17 (3.11%)	69 (12.64%)	25 (4.76%)	4 (0.73%)	3 (0.55%)	4 (0.73%)	41 (7.51%)	126 (23.08%)	50 (9.16%)	15 (2.75%)	19 (3.48%)	1 (0.18%)	28 (5.13%)	92 (16.85%)	25 (4.58%)	13 (2.38%)	12 (2.20%)	1 (0.18%)
T2DM with Heart Failure	12 (2.20%)	62 (11.36%)	27 (4.95%)	6 (1.10%)	10 (2.01%)	3 (0.55%)	54 (9.89%)	174 (32.05%)	55 (10.07%)	18 (3.30%)	17 (3.11%)	2 (0.37%)	20 (3.66%)	50 (9.16%)	19 (3.48%)	7 (1.47%)	6 (1.10%)	1 (0.18%)
